# High proportions of foods recommended for consumption by United States Dietary Guidance contain solid fats and added sugar: results from the National Health and Nutrition Examination Survey (2007-2008)

**DOI:** 10.1186/1475-2891-13-23

**Published:** 2014-03-20

**Authors:** Lisa Jahns, Sibylle Kranz

**Affiliations:** 1 Research Nutritionist, United States Department of Agriculture, Agricultural Research Service, Grand Forks Human Nutrition Research Center, 2420 2nd Ave N, Grand Forks, ND 58203, USA; 2 Department of Nutrition Science, College of Health and Human Sciences, Purdue University, 204 Stone Hall, 700W. State Street, West Lafayette, IN 47907, USA

**Keywords:** Solid fats, Added sugars, WWEIA, NHANES, Diet quality

## Abstract

**Background:**

The 2010 Dietary Guidelines for Americans (DGA) recommend that individuals age two years and older reduce intakes of solid fats (SoF) and added sugars (AS; together SoFAS). MyPlate illustrates the proportions of five major food groups to promote healthy eating (Vegetables, Grains, Protein Foods, Fruits and Dairy).

**Methods:**

To assess if the foods currently consumed by Americans are in concordance with the DGA, food consumption data from What We Eat In America, National Health and Nutrition Examination Survey (WWEIA-NHANES) 2007–2008 (n = 8 527) was used to estimate the proportion of foods that contained SoFAS and to report them by food group. Weighted analysis was conducted to be nationally representative.

**Results:**

The Dairy group contained the highest proportion (93%) of either SoF or AS, followed by Grains (70% SoF; 70% AS; 50% both). Fruits contained the least SoFAS (7%).

**Conclusions:**

Results suggest that the high proportion of SoFAS in each recommended food group makes it challenging for Americans to reduce their intake of SoFAS.

## Background

In the United States (US), people of all ages fail to meet federal dietary intake recommendations for a healthy diet; in particular, the consumption of fruits, vegetables, and whole grains is suboptimal [1]. Although recommendations have consistently encouraged intake of these food groups to promote healthy eating [2,3], recent considerations of the changes in the US food energy supply have shown that people eat more food [4], but not more healthfully than in the past. Dietary guidance to consumers must be simple and direct; therefore, it is difficult to convey a comprehensive message regarding the complex and ever-changing food supply [5,6].

The intent of the 2010 Dietary Guidelines for Americans (DGA) is to present a set of recommendations for consumers aged two years and older to encourage healthy eating. The DGA specifically recommends “Reduce the intake of calories from solid fats and added sugars” [3]. These recommendations and suggestions for food groups to consume either more or less of were distilled into the national dietary guidance icon “MyPlate” [7] to provide easy-to-follow consumer targeted guidelines.

Based on the definitions used to describe the food consumption by the American people, the following criteria to determine solid fats and added sugars are: “solid fats, any fat that is solid at room temperature (which includes naturally-occurring fat found in full-fat milk and yoghurt, fatty cuts of meat and butter and spreads), and added sugars, (non-naturally occurring sugars added to food, such as sodas and candies), are also known as “empty calories” as they provide little or no nutrients yet are high in calories” [7]. While most non-US food intake studies do not differentiate between added and naturally occurring sugars, the US uses the term “added sugars” to imply the level of control the consumer has over consuming these types of sugars. Since a healthy diet with large proportions of fruits and dairy (consequently high in fructose and lactose) can be very high in total sugars (depending on the products chosen), the amount of added sugars consumed is a direct measurement of the consumer’s preference for highly sweetened foods. For instance, a cup of non-sweetened cereal with pineapple results in very low values for added sugars, however, the same cereal with a tablespoon of sucrose (household sugar), results in 3 tsp of added sugar – although the food itself might be perceived as having the same level of sweetness. Dietary fats that are solid at room temperature (such as lard or butter), are naturally high in saturated fats and therefore considered less desirable to support a healthy diet. Foods high in unsaturated fats, such as olive or safflower oil are liquid at room temperature. Thus, the use of the term “solid fats” is a proxy for “high saturated fats”.

Since the DGA and MyPlate recommendations are based on the total diet, not individual foods, no maximum amounts of solid fats and added sugars (SoFAS) for any given food are provided. Thus, consumers may choose to consume eat any combination of items from food groups, and - at least in theory - still achieve a healthy diet. Britten et al. modeled the effects on the total diet meeting the DGA when foods low in SoFAS were chosen instead of the usual American diet. They found that the guidelines can be met, but only with a large increase in the choice of low-fat, no added sugar foods [8]. Other groups, using the same data, suggest that higher amounts of SoFAS should be allowed [9,10]. These reports focus on food group consumption by individuals, whilst this research focuses on food level. These results may be used to inform future guidance to consumers that is simple to follow yet helpful when attempting to navigate a complicated food supply. The aim of the present study was to better understand the potential implications of following the DGA and MyPlate recommendations in the context of the public’s food choices. Dietary intake data from What We Eat In America, National Health and Nutrition Examination Survey (WWEIA-NHANES) 2007–2008 (n = 4 046 foods) was used to estimate the proportion of foods that contain either SoF, or AS in the major MyPlate food groups (Vegetables, Grains, Protein Foods, Fruits and Dairy) as well as two additional groupings of public health significance (Fats & Oils and Sugars, Sweets, & Beverages). Results were weighted to reflect nationally representative intake. The proportion of the foods containing SoFAS was described stratified for each of the seven food groups and results discussed in the context of the complexity of consumer messaging of federal dietary guidance.

## Methods

The NHANES protocol was approved by the National Center for Health Statistics Research Ethics Review Board and all participants provided informed consent.

Data were obtained from the United States Department of Agriculture, Agricultural Research Service’s (USDA-ARS) 2007–2008 What We Eat in America (WWEIA) survey, which comprises the dietary intake component of the National Health and Nutrition Examination Survey (NHANES) 2007–2008. The WWEIA survey consists of two non-consecutive 24-hr recalls; the first completed in-person and the second by phone. For the purposes of this study, foods reported in the first recall by 8 528 individuals two years and older were included. Individuals age 12 and over reported their own diet and caregivers provided and assisted in recalls by children aged 2–12. Complete information on WWEIA can be found elsewhere [11]. The total food consumption files include all single ingredient as well as composite foods and mixed dishes that were reported by the survey respondents [12] First, to categorize food amounts into the five MyPlate and two additional food groups, the USDA-ARS MyPyramid Equivalents Database for 2.0 for USDA Survey Foods, 2003–2004 [13] and its supplement (released March 2012) was employed. The MPED database was specifically designed to translate the amounts of foods reported by participants in the WWEIA into equivalent servings of the 32 MyPyramid major and sub groups, including “Discretionary solid fat” and “Added sugars”. Although the simpler MyPlate icon has replaced the MyPyramid food guidance icon, the major food groups are identical.

Each food description is linked to a 8-digit food code, where the first digit identifies one of the nine major food groups (1 = Milk and Milk Products, 2 = Meat, Poultry, Fish, and Mixtures, 3 = Eggs, 4 = Dry Beans, Peas, Other Legumes, Nuts, and Seeds, 5 = Grain Products, 6 = Fruits, 7 = Vegetables, 8 = Fats, Oils, and Salad Dressings, 9 = Sugars, Sweets, and Beverages), the second digit indicates subgroups within each major food group and mixed foods, and the third and subsequent digits provide ever-more detailed discriminations until the level of individual food items. For this analysis, food codes were grouped by the primary level of coding to ascertain intake of the main MyPlate food groups, Vegetables, Grains, Protein Foods, Fruits and Dairy. Groups 2, 3, and 4 were combined into the Protein Foods group. Foods categorized as Fats, Oils and Salad Dressings and Sugars, Sweets, and Beverages group are not part of the MyPlate recommendations, but are of public health significance and were therefore presented separately. The Fats, Oils and Salad Dressing group includes fats that are solid at room temperature (SoF), including naturally-occurring fats, mostly from animal products, and hydrogenated vegetable oils. It also includes solid fats, such as butter and solid margarine that are added at the discretion of the consumer. Oils include naturally-occurring oils such as those nuts and seeds, non-hydrogenated vegetable oils, salad dressings, and soft margarines. The Sugars, Sweets and Beverage group includes candies, sweet desserts, sugar added at the table at the table, sugar-sweetened beverages, water, tea and coffee. Sugar from fruits is not included.

As the DGA recommendations pertain to individuals aged two and over, baby foods, formula and breast milk were excluded, resulting in an analytical sample of 4 046 unique foods reported by 8 527 respondents a total of 135 934 times. All foods consumed by the sample population were categorized as containing SoF (=1), AS (=1) or both (=2). We also include a category of “either or both” as many foods contain both SoF and AS. Tertiles of SoF and AS in each category are presented to show an approximate range of values within food group categories. The amount of SoFAS in each food was not calculated as no specific maximum recommended amount of SoFAS in any given food has been established. For instance, a dairy food may have 10 grams of AS, which would be considered excessive for a person who consumes many other foods high in AS, but could be an appropriate addition to a healthy diet for a person consuming few other high-AS foods. Thus, in contrast to other studies examining the sources of SoFAS at the level of individual diet, this study provides an overview of food choice at the aggregate level. Sampling weights were used to adjust estimated results to maintain the nationally representative character of the data. Weighted proportions of the foods in each of the food groups and their SoF, AS, and SoFAS presence were calculated.

## Results

Overall, 60% of foods consumed contained SoFAS, or empty calories, of those, 43% contained SoF and 39% contained AS, 22% contained both. Disaggregation of the foods consumed into MyPlate food groups are presented in Table 1.

**Table 1:** Weighted* proportion of the prevalence of SoFAS in foods reported in the 2007–2008 WWEIA-NHANES survey (n = 8 527) by MyPlate food groupings

	**Percent (%) of foods in each group**			
**MyPlate food groups**	**Added sugar**	**Solid fat**	**Both SoFAS**	**Either SoFAS or both SoFAS**
Vegetables	19.6	27.3	8.8	38.0
Grains	70.2	69.1	50.2	89.2
Protein foods	31.4	73.2	27.3	77.3
Fruits	6.8	0.5	0.4	6.8
Dairy	30.0	92.3	29.6	92.7
**Other food groups**	**Added sugar**	**Solid fat**	**Both SoFAS**	**Either SoFAS or both SoFAS**
Fats & oils	54.3	67.5	41.2	80.6
Sugars, sweets, beverages	38.3	6.1	5.7	38.7

*The WWEIA-NHANES survey contains weights based off of the US Census; when applied, the estimated results of the survey are adjusted to be representative of total US population.

Over two-thirds of the foods in the Vegetables group contained either SoF or AS compared to less than 10% of fruits, derived mostly from AS. Nearly three-fourths of the Grains, Protein, and Dairy groups contained SoF; including over 90% of Dairy foods. Half of the foods in the Grains group contained either or both SoFAS with roughly equal amounts contributed by SoF and AS. Half of the foods in the Fats and Oils group included AS and over two-thirds contained SoF. Although the Sugars, Sweets, and Beverages group might be expected to have large proportions of foods with SoFAS, the inclusion of non-sweetened water, tea, or coffee constituted over 50% of this group (data not shown); therefore, the foods consumed from this group had a relatively small proportion of foods containing SoFAS. Although there are no recommended amounts of SoFAS, tertiles of SoF (g/100 g) and AS (tsp) content of foods in each group are presented in Tables 2 and 3, respectively. Most of the SoF reported in the Grains, Protein Foods, and Dairy groups came from foods in the highest tertile of SoF. Again, most of the AS in the Dairy group came from foods in the highest tertile of AS, while the Protein Foods and Vegetables groups derived most AS from the lowest tertile. Many reported foods, however, fell into the middle tertile of AS.

**Table 2:** Tertiles of the weighted* proportion of the solid fat (SoF [g/100 g] food) in foods reported in the 2007–2008 WWEIA-NHANES survey (n = 8 527) by MyPlate food groupings

**MyPlate food group**	**None**	**Low SoF % (> 0 SoF ≤ 2.5)**	**Middle SoF % (0.25 < SoF < 8.8)**	**Higest SoF % (SoF ≥ 8.8)**
Vegetables	72.7	11.0	7.2	9.0
Grains	30.9	15.2	24.1	29.8
Protein foods	26.8	21.1	22.2	30.0
Fruits	99.6	0.2	0.26	0.02
Dairy	7.7	30.3	14.8	47.2
**Other food groups**				
Fats & oils	32.5	40.0	2.6	24.9
Sugars, sweets, beverages	93.9	1.4	2.3	2.3

*The WWEIA-NHANES survey contains weights based off of the US Census; when applied, the estimated results of the survey are adjusted to be representative of total US population.

**Table 3:** Tertiles of the weighted* proportion of the added sugar (AS [tsp]) in foods reported in the 2007–2008 WWEIA-NHANES survey (n = 8 527) by MyPlate food groupings

**MyPlate food group**	**None**	**Low AS % (> 0 AS ≤ 0.52)**	**Middle AS % (0.52 < AS < 3.5)**	**Higest AS % (AS ≥ 3.5)**
Vegetables	80.4	9.5	2.8	7.3
Grains	29.8	14.5	32.5	23.2
Protein foods	68.7	19.6	11.3	0.4
Fruits	93.2	0.2	5.4	1.2
Dairy	70.0	1.8	13.8	14.4
**Other food groups**				
Fats & oils	45.7	17.6	33.0	3.66
Sugars, sweets, beverages	61.7	0.5	21.1	16.7

*The WWEIA-NHANES survey contains weights based off of the US Census; when applied, the estimated results of the survey are adjusted to be representative of total US population.

## Discussion

The majority of foods consumed within the MyPlate food grouping system contained SoFAS. Almost all of the foods in the Dairy group contain either SoF or AS, mostly in the form of SoF and over three-fourths of the foods in the Grains and Protein Foods groups contained either AS or SoF. Within the categories of foods containing either SoF or AS, tertiles of the amounts of either show that the frequency of consumption of foods in these groups often does not consist of foods that fall into the highest tertiles of SoF or AS content, highlighting the difficulty of choosing foods low in SoF or AS. However, no absolute value of the SoF or AS tertiles may be considered “good” or “bad” foods given the lack of specific cut-off amounts allowed in a healthy diet independent of energy needs.

Although the amounts of SoFAS in the foods may vary, consumption of large numbers of foods containing these components predicts low diet quality and excess energy intake [8], although others have suggested that foods high in SoF or AS contribute to nutrient intakes and suggest that advice to reduce these foods could lower diet quality [10]. Other literature has reported the food sources of SoFAS and energy intake at the individual level and found that many of the foods that contributed the most energy to children’s diets, such as pizza, soda, and full-fat milk, all contained SoFAS [14]. The analysis presented here was designed to examine the type of food consumed on the food group level, thus, single foods, individual diet quality or the daily amount of SoFAS consumed was not examined. Additional factors contributing to diet quality, such as sodium content or the nutrient and/or energy density of foods were also not included - although their importance was established in the DGA 2010 recommendations. The DGA 2010 makes 7 key recommendations for “foods and food components to reduce”, one of which is “reduce the intake of calories from solid fats and added sugars”. We have chosen to focus on this single guideline as these components are often difficult to discern in foods. This is the first time the guidelines have suggested reducing, instead of limiting, intake of SoFAS, making the identification of the prevalence of these components in recommended food groups of special interest.

Limitations of this study include that the analysis was based on self-reported dietary intake data. However, the major strength of this analysis was that the researchers did not impose any judgment based on questions such as the potential value of consuming foods despite the presence of SoFAS in the food. For instance, flavoured milk usually has added sugar and therefore might be considered a food that should be limited. However, one might argue against limiting flavoured milk intake because many people notoriously under-consume calcium-rich foods and might therefore benefit from the drinking flavoured milk - especially if the flavoured milk were to replace soda or sweetened fruit drinks/ades [10,15]. An additional strength of this analysis was the nationally representative character of the data and the use of recent information on the US diet.

The classification of foods into either having SoFAS or not, removed any potential bias towards certain foods within any of the five MyPlate food groups. Future research might include analysis in population sub-groups to establish population groups at highest risk for consuming foods with SoFAS. In addition, examination of the average amount of SoFAS in the foods within each MyPlate category might provide a venue for improved dietary guidance for the population.

Diet patterns usually establish over time and reflect typical choices or food intake habits. While the use of mathematical modelling to create disease-specific prevention food plans or patterns, such as a diet plan to prevent cancer [16], might be an approach helpful to some people, the general population might benefit more from information on the food level to better understand implications of various types of food processing and packaging during the process of making food intake choices. Due to the large use of commercially available foods, which may include ingredients to maximize flavour and increase the desirability of foods, it might be too complex for the average American to discern the healthier from the less healthy food choices. In addition, food preparation and serving methods, such as the amount of fat added to vegetables at mealtime, might be underestimated. These factors considerably influence the nutritional quality of the foods chosen by the public [17].

## Conclusions

This analysis of nationally representative US food consumption data revealed a surprisingly high proportion of foods consumed with SoFAS, or empty calories. Establishing guidelines for maximum values of the SoF or AS content of a food, similar to the percent daily values, might be a venue to help people limit those two food components. The approach used here represents a new and critically important way of examining American’s adherence to federal dietary guidance. Similar analysis in assessing or planning population-based food consumption education could help policy makers and health professionals to target the foods with the highest potential to lead to a low quality diet.

## Abbreviations

DGA: Dietary Guidelines for Americans; SoF: Solid fats; AS; together SoFAS: Added sugars; WWEIA-NHANES: What we eat in America, national health and nutrition examination survey; MPED 2.0: MyPyramid equivalents database for 2.0 for USDA survey foods, 2003–2004; USDA: United States Department of Agriculture; FNDDS: Food and nutrient database for dietary studies; SR: Standard reference.

## Competing interests

The authors declare that they have no competing interests.

## Authors’ contributions

LJ and SK jointly conceived and designed the experiment, analysed the data, wrote the paper and critically reviewed it. Both authors read and approved the final manuscript.

## References

[B1] Krebs-Smith SM Guenther PM Subar AF Kirkpatrick SI Dodd KW Americans do not meet federal dietary recommendations J Nutr 1832–1838 2010 140 10.3945/jn.110.124826 PMC2937576 20702750

[B2] U.S. Department of Health and Human Services and U.S. Department of Agriculture Dietary Guidelines for Americans, 2005 2005 6 Washington, DC: U.S. Government Printing Office

[B3] US Department of Agriculture and US Department of Health and Human Services Dietary Guidelines for Americans, 2010 2010 7 Washington, DC: US Government Printing Office

[B4] Swinburn B Sacks G Ravussin E Increased food energy supply is more than sufficient to explain the US epidemic of obesity Am J Clin Nutr 2009 90 1453 1456 10.3945/ajcn.2009.28595 19828708

[B5] Krebs-Smith SM Reedy J Bosire C Healthfulness of the U.S. food supply: little improvement despite decades of dietary guidance Am J Prev Med 2010 38 472 477 10.1016/j.amepre.2010.01.016 20153133 PMC2858769

[B6] Reedy J Krebs-Smith SM Bosire C Evaluating the food environment: application of the Healthy Eating Index-2005 Am J Prev Med 2010 38 465 471 10.1016/j.amepre.2010.01.015 20171823

[B7] US Department of Agriculture ChooseMyPlate http://www.choosemyplate.gov/

[B8] Britten P Clevelnad LE Koegel KL Kuczynski KJ Nickols-Richardson SM Impact of typical rather than nutrient-dense food choices in the US Department of Agriculture food patterns J Acad Nutr Diet 2012 112 1560 1569 10.1016/j.jand.2012.06.360 22906562

[B9] Maillot M Drewnowski A Energy allowances for solid fats and added sugars in nutritionally adequate U.S. diets estimated at 17-33% by linear programming J Nutr 2011 141 333 340 10.3945/jn.110.131920 21178090 PMC3021454

[B10] Huth PJ Fulgoni VL Keast DR Park K Auestad N Major food sources of calories, added sugars, and saturated fat and their contribution to essential nutrient intakes in the U.S. diet: data from the national health and nutrition examination survey (2003–2006) Nutr J 2013 12 116 10.1186/1475-2891-12-116 23927718 PMC3751311

[B11] US Department of Agriculture, Agricultural Research Service, Beltsville Human Nutrition Research Center, Food Surveys Research Group (Beltsville, MD) What We Eat in America, NHANES 2007–2008 Data: Dietary Interview - Total Nutrients Intakes -- First Day (DR1IFF_E) 2013 http://www.cdc.gov/nchs/about/major/nhanes/nhanes2007-2008/dr1tot_c.xpt

[B12] United States Department of Agriculture USDA Food and Nutrient Database for Dietary Studies 4.1 2010 Beltsville, MD: Agricultural Research Service, Food Surveys Research Group http://www.ars.usda.gov/Services/docs.htm?docid=20511

[B13] Bowman SA Friday JE Moshfegh A MyPyramid Equivalents Database, 2.0 for USDA Survey Foods, 2003–2004 2008 Beltsville, MD: Food Surveys Research Group. Beltsville Human Nutrition Research Center, Agricultural Research Service, US Department of Agriculture http://www.ars.usda.gov/ba/bhnrc/fsrg

[B14] Reedy J Krebs-Smith SM Dietary sources of energy, solid fats, and added sugars among children and adolescents in the United States J Am Diet Assoc 2010 110 1477 1484 10.1016/j.jada.2010.07.010 20869486 PMC3428130

[B15] Livingstone MBE Rennie KL Added sugars and micronutrient dilution Obes Reviews 2009 10 Suppl. 1 34 40 10.1111/j.1467-789X.2008.00563.x 19207534

[B16] Masset G Monsivais P Maillot M Darmon N Drewnowski A Diet optimization methods can help translate dietary guidelines into a cancer prevention food plan J Nutr 2009 139 1541 1548 10.3945/jn.109.104398 19535422

[B17] Rowe S Alexander N Almeida NG Black R Burns R Bush L Crawford P Keim N Kris-Etherton P Weaver C Translating the Dietary Guidelines for Americans 2010 to bring about real behavior change J Am Diet Assoc 2011 111 28 39 10.1016/j.jada.2010.11.007 21185961

